# Apoptosis-dependent head development during metamorphosis of the cnidarian *Hydractinia symbiolongicarpus*

**DOI:** 10.1016/j.ydbio.2024.08.010

**Published:** 2024-08-18

**Authors:** Gabriel Krasovec, Uri Frank

**Affiliations:** 1 Centre for Chromosome Biology, School of Biological and Chemical Sciences, University of Galway, Galway, Ireland

**Keywords:** Apoptosis, Caspase, Cell differentiation, Metamorphosis, Nerve cells

## Abstract

Apoptosis is a regulated cell death that depends on caspases. It has mainly been studied as a mechanism for the removal of unwanted cells. However, apoptotic cells can induce fate or behavior changes of their neighbors and thereby participate in development. Here, we address the functions of apoptosis during metamorphosis of the cnidarian *Hydractinia symbiolongicarpus*. We describe the apoptotic profile during metamorphosis of the larva and identify *Caspase3/7a*, but no other executioner caspases, as essential for apoptosis in this context. Using pharmacological and genetic approaches, we find that apoptosis is required for normal head development. Inhibition of apoptosis resulted in defects in head morphogenesis. Neurogenesis was compromised in the body column of apoptosis-inhibited animals but there was no effect on the survival or proliferation of stem cells, suggesting that apoptosis is required for cellular commitment rather than for the maintenance of their progenitors. Differential transcriptomic analysis identifies TRAF genes as downregulated in apoptosis-inhibited larvae and functional experiments provide evidence that they are essential for head development. Finally, we find no major role for apoptosis in head regeneration in this animal, in contrast to the significance of apoptosis in *Hydra* head regeneration.

## Introduction

Apoptosis is a regulated cell death, defined by morphological features and dependence on caspases (Hengartner, 2000; Kerr et al., 1972). Widely present in Metazoa (Abdelwahid et al., 2007; Böttger and Alexandrova, 2007; Krasovec et al., 2019, 2021b, 2024; Lockshin and Williams, 1964; Sokolova et al., 2004; Vega Thurber and Epel, 2007), apoptosis is crucial not only for eliminating unwanted cells in development and homeostasis (Jacobson et al., 1997), but also controls the fate and behavior of live cells (Fogarty and Bergmann, 2015; Krasovec et al., 2022, 2021a). For example, apoptotic cells can induce proliferation during regeneration in the cnidarian *Hydra* (Chera et al., 2009). In mammals, apoptotic cells attract immune cells leading to the clearance of apoptotic bodies (Lauber et al., 2003). The functions of apoptosis have been mainly studied in mammals. Therefore, investigations at a broader phylogenetic scale are required to understand the evolution of apoptosis and its ancestral functions (Horkan et al., 2023; Krasovec et al., 2023, 2022). Cnidarians, as the sister group of bilaterians, offer a unique opportunity to understand apoptotic functions in animals (Frank et al., 2001).

Here, we address the role of apoptosis during metamorphosis of the larva in the cnidarian *Hydractinia symbiolongicarpus. Hydractinia* is a clonal and colonial animal, presenting a bi-phasic life cycle (Frank et al., 2020). The settled colony is composed of feeding and sexual zooids called polyps, sharing a gastrovascular network called stolons that are attached to the substratum. Polyps are structured as a cylindrical column with a mouth surrounded by tentacles at the one pole (the oral pole). They are connected to the stolonal network at the opposite pole (the aboral pole). A continuous network of neurons innervates the entire colony (Chrysostomou et al., 2022b). Sexual polyps release gametes directly into the water where embryogenesis commences, producing a motile planula larva within 3 days. Upon receiving an appropriate environmental signal, the larva settles on the substratum and metamorphoses into a primary polyp, the founding member of a new colony (Frank et al., 2020; Leitz, 1998; Müller and Leitz, 2002). Apoptosis, controlled by a *Caspase-3*-like gene, has been shown to play an important role in the metamorphosis of *H. echinata* (Seipp et al., 2001). We have expanded the knowledge on apoptosis in the sister species, *H. symbiolongicarpus*. Pharmacological and genetic inhibition of all predicted *Hydractinia* executioner caspases identified only one caspase gene, *Caspase3/7a*, as essential for the apoptotic event in early metamorphosis. Apoptosis inhibition induced defects in tentacle morphogenesis and inhibition of neurogenesis in the head and body column. Knocking down of two TRAF genes that were differentially expressed following metamorphosis induction phenocopied the inhibition of apoptosis. These genes probably act downstream apoptosis in this context.

## Results and discussion

### The *H. symbiolongicarpus* genome encodes both initiator and executioner caspases

**Figure 1:**
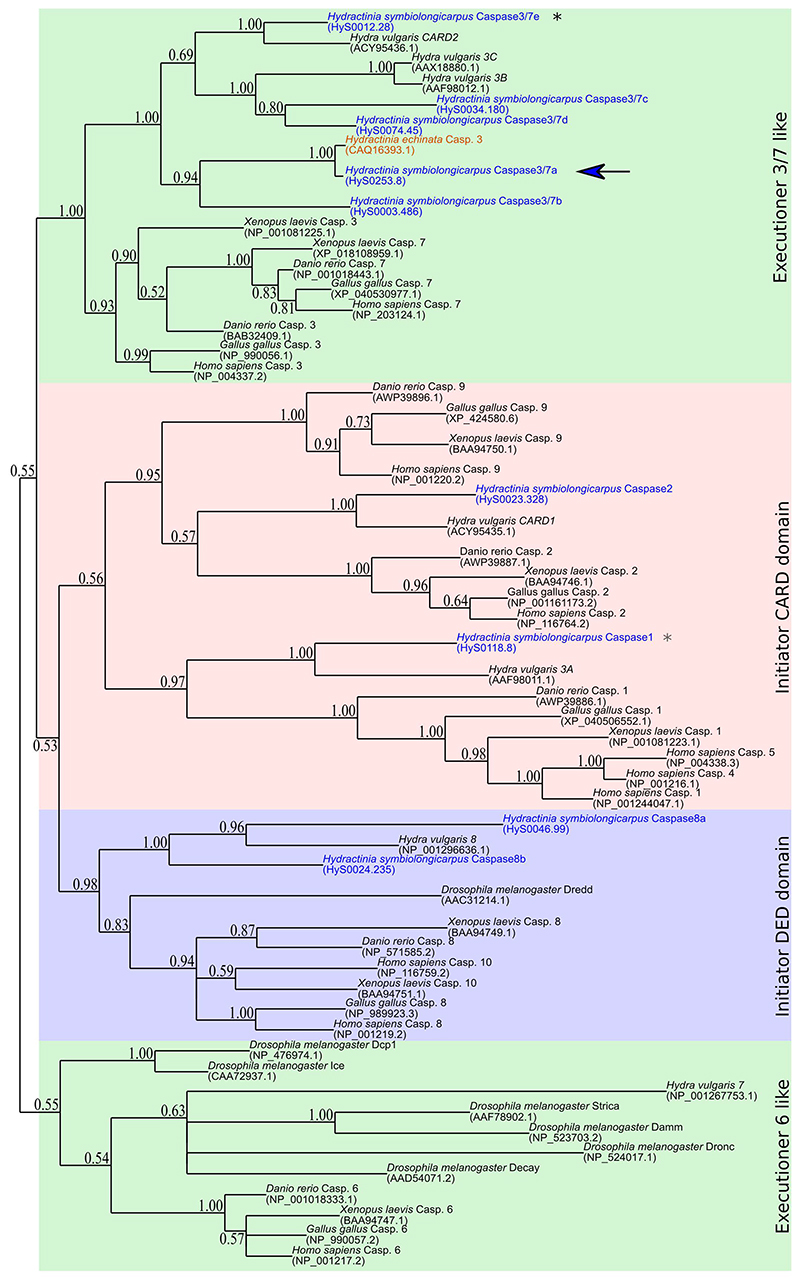
Phylogeny of caspases obtained by Bayesian inference. The *Hydractinia symbiolongicarpus* caspase family (gene names in blue) is composed of nine caspases, including two with a CARD domain and two others with a DED domain. *H. symbiolongicarpus Caspase3/7a* (arrow) is orthologue to *Hydractinia echinata* caspase 3. *H. symbiolongicarpus Caspase1* (grey*), devoid of CARD prodomain, is localized inside the initiator caspases cluster, but sister to *Hydra* caspase 3A. *H. symbiolongicarpus Caspase3/7e* (black*), which has a CARD prodomain, clusters with vertebrate executioner caspases.

Members of the caspase multigenic family are central in apoptosis regulation (Hengartner, 2000). Caspases are divided into initiators, playing a role at the onset of the signaling pathway, and executioners that conduct the cell death. Initiator caspases are characterized by the presence of a long prodomain that can be a CARD (CARD-caspase) or a DED (DED-caspase) domain, while executioner caspases only have the P10 and P20 domains, shared by the whole family (Fan et al., 2005; Kumar, 2007). The *H. symbiolongicarpus* genome encodes 9 caspases (Kon-Nanjo et al., 2023) (see Table S1 for gene IDs in the two available genome assemblies), including 2 CARD-caspases and 2 DED-caspases, and five caspases devoid of a long prodomain; the latter having the architecture of classical executioner caspases (Figure 1). Phylogenetic analysis identified four clusters, which represent the executioner caspases 6 (0.55 posterior probability – PP), the CARD-caspases (0.56 PP), the DED-caspases (0.98 PP), and the executioner caspases 3 and 7 groups (1.00 PP) (Figure 1). The two *H. symbiolongicarpus* DED-caspases (*Caspase8a* and *Caspase8b*) clustered, as expected, within the DED-caspases clade. *Caspase2*, having a CARD domain, was grouped with the other CARD-caspases together with *Hydra* CARD1, both related to vertebrate caspase 2. No caspase 9 was found in *H. symbiolongicarpus*, consistent with our previous work showing that this initiator is deuterostome-specific (Krasovec et al., 2023). Notably, *Caspase1*, closely related to the inflammatory caspases of vertebrates, is devoid of a CARD domain and is orthologous to the *Hydra* executioner caspase-3A (Lasi et al., 2010b) (Figure 1). *H. symbiolongicarpus* does only encode five executioner caspases, *Caspase3/7a, Caspase3/7b, Caspase3/7c, Caspase3/7d*, and *Caspase3/7e*, which are homologous to caspases 3 and 7 of vertebrates. *H. symbiolongicarpus Caspase3/7e*, which has a CARD domain, is orthologous to *Hydra vulgaris* caspase CARD2 (Cikala et al., 1999; Lasi et al., 2010a) within the caspases 3 and 7 clade.

### Apoptosis accompanies metamorphosis in *H. symbiolongicarpus*

**Figure 2:**
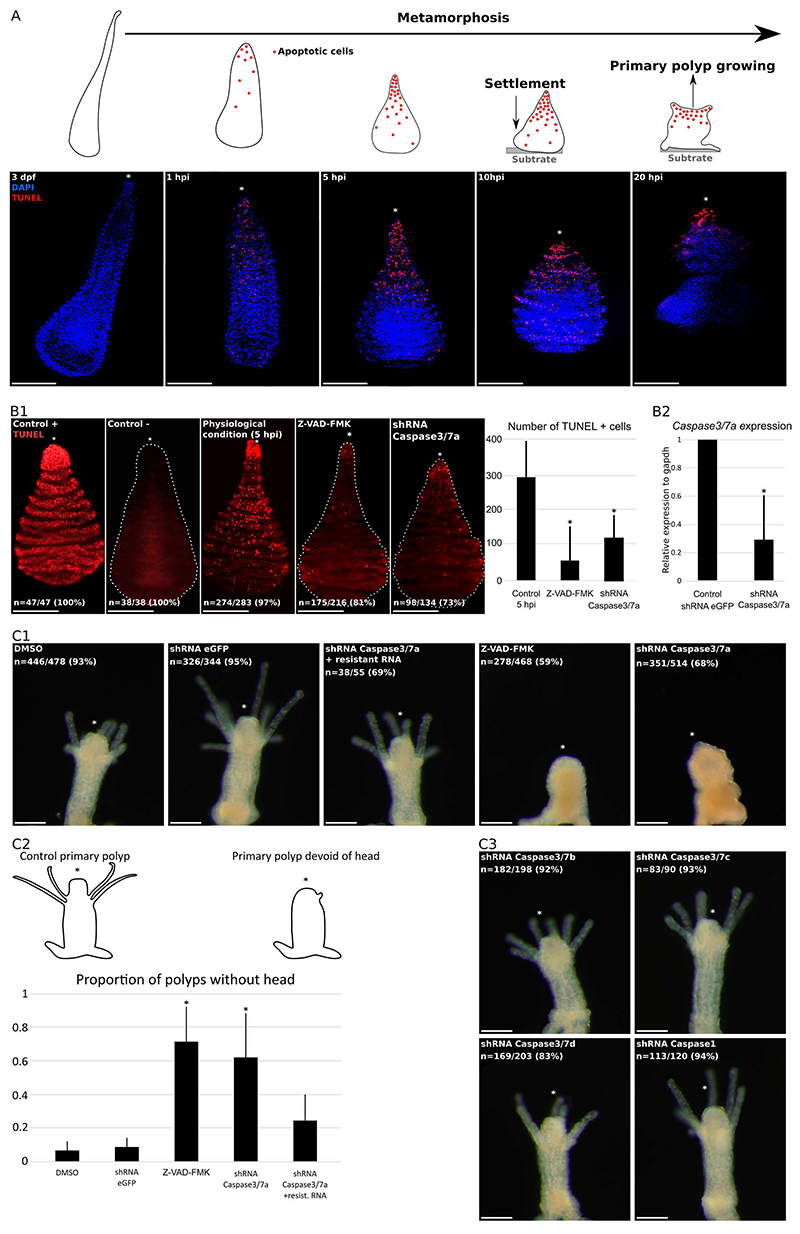
Apoptosis is required for head morphogenesis during *Hydractinia* metamorphosis. **A**, Apoptosis starts about 1 hour post-induction (hpi) and is restricted to the epidermis. Apoptosis is polarized with most TUNEL^+^nuclei detected in the oral pole. **B**, The number of TUNEL^+^nuclei usually detected at 5 hpi under physiological condition is significantly reduced by the Z-VAD-FMK treatment and the *Caspase3/7a* shRNA injection (B1). Real-time PCR confirmed the efficiency of *Caspase3/7a* shRNA which reduced the amount of *Caspase3/7a* mRNA (B2). Positive control was treated with DNase 1. Negative controls were incubated in TUNEL reaction mix devoid of terminal deoxynucleotidyl transferase (tdt enzyme). **C1**, Control primary polyps (DMSO, shRNA eGFP injected) developed normally. Pan-caspases inhibitor Z-VAD-FMK and *Caspase3/7a* knockdown affected head phenotype, resulting in polyps with stunted heads, devoid of tentacles. Injection of a *Caspase3/7a* resistant mRNA together with the shRNA rescued the phenotype. **C2**, Effect of apoptosis disruption on head development is statistically significant. **C3**, Loss of function of *Caspase3/7b* to *Caspase3/7d* and *Caspase1* had no visible phenotype. Dfp: days post-fertilisation. Hpi: hours post-induction. A and B, scale bar = 100µm. C, scale bar = 200µm. * in polyps images: oral pole. * in graph: p-value<0.05.

*Hydractinia* metamorphosis is characterized by contraction of the larva along the oral-aboral axis, followed by the attachment to a substratum, and the formation of a flattened structure from which the primary polyp develops (Müller and Leitz, 2002). The larval anterior pole (aboral pole) becomes the stolon while the tapered tail develops into the head (oral pole). Apoptosis in *H. symbiolongicarpus* starts during the first hour post-metamorphosis induction (hpi) similar to *H. echinata* (Leitz, 1998; Seipp et al., 2007, 2001), and TUNEL^+^ nuclei remain visible until primary polyp growth (Figure 2A). The apoptotic profile is spatially polarized with a high concentration of TUNEL^+^ nuclei in the epidermis of the oral pole (i.e., the larval tail) (Figure 2A), consistent with the observations made in *H. echinata* (Seipp et al., 2006, 2001). Only few TUNEL^+^ nuclei could be detected at the aboral epidermis in *H. symbiolongicarpus*, while in *H. echinata*, apoptotic cells form a ring at the aboral pole (Seipp et al., 2006). Interestingly, we never detected gastrodermal apoptosis in *H. symbiolongicarpus*, in contrast to what was reported in *H. echinata*. This observation persisted even after 4 hours of permeabilization, and increasing the incubation time in the TUNEL mixture to 90 min.

### Inhibition of apoptosis prevents proper head development during metamorphosis

It has been shown that *H. echinata* metamorphosis is caspase-dependent (Seipp et al., 2006). Treating the larvae with the pan-caspase inhibitor Z-VAD-FMK, or knocking-down *He-caspase-3*, prevents metamorphosis (Seipp et al., 2006; Wittig et al., 2011). We confirmed the effect of Z-VAD-FMK in *H. symbiolongicarpus* and focused, in addition, on all predicted executioner caspases encoded in the *Hydractinia* genome, as these are the ones responsible for the final apoptosis execution (Figure 2B-C) (Cohen, 1997; Hengartner, 2000; Kumar, 2007). *H. symbiolongicarpus Caspase3/7a* is orthologous to the executioner caspase He-caspase-3, identified to be crucial for metamorphosis of *H. echinata* (Figure 1) (Wittig et al., 2011). shRNA-mediated knockdown of *Caspase3/7a* showed that it is indispensable for the apoptotic event at the onset of metamorphosis, and for proper head development (Figure 2C). We then tried do disrupt other caspases with a domain architecture of executioner caspases. Loss of function of *Caspase3/7b to Caspase3/7d*,and *Caspase1*did not inhibit apoptosis, nor did it affect metamorphosis (Figure 2C). The specificity of the *Caspase3/7a* shRNA was confirmed by rescue experiments using co-injection of the shRNA and *Caspase3/7a* mRNA that was rendered resistant to the shRNA by three silent mutations (Figure 2C, Figure S1).

### Apoptosis inhibition interferes with neurogenesis

The *Hydractinia* head is composed of various cell types, including but not limited to epithelia and neural cells; the latter comprise neurons and stinging cells (nematocytes). Differentiating nematocytes (i.e., nematoblasts) are located in the body column and can be detected by Ncol1 or Ncol3 immunostaining (Chrysostomou et al., 2022b; Kanska and Frank, 2013), while lectin labelling allows detection of fully differentiated nematocytes that are mainly, but not exclusively, located in the tentacles (Bradshaw et al., 2015). Neurons, which can be visualized by RFamide immunostaining (Chrysostomou et al., 2022b; Kanska and Frank, 2013), form a network in the body column with a higher density in the head region.

**Figure 3:**
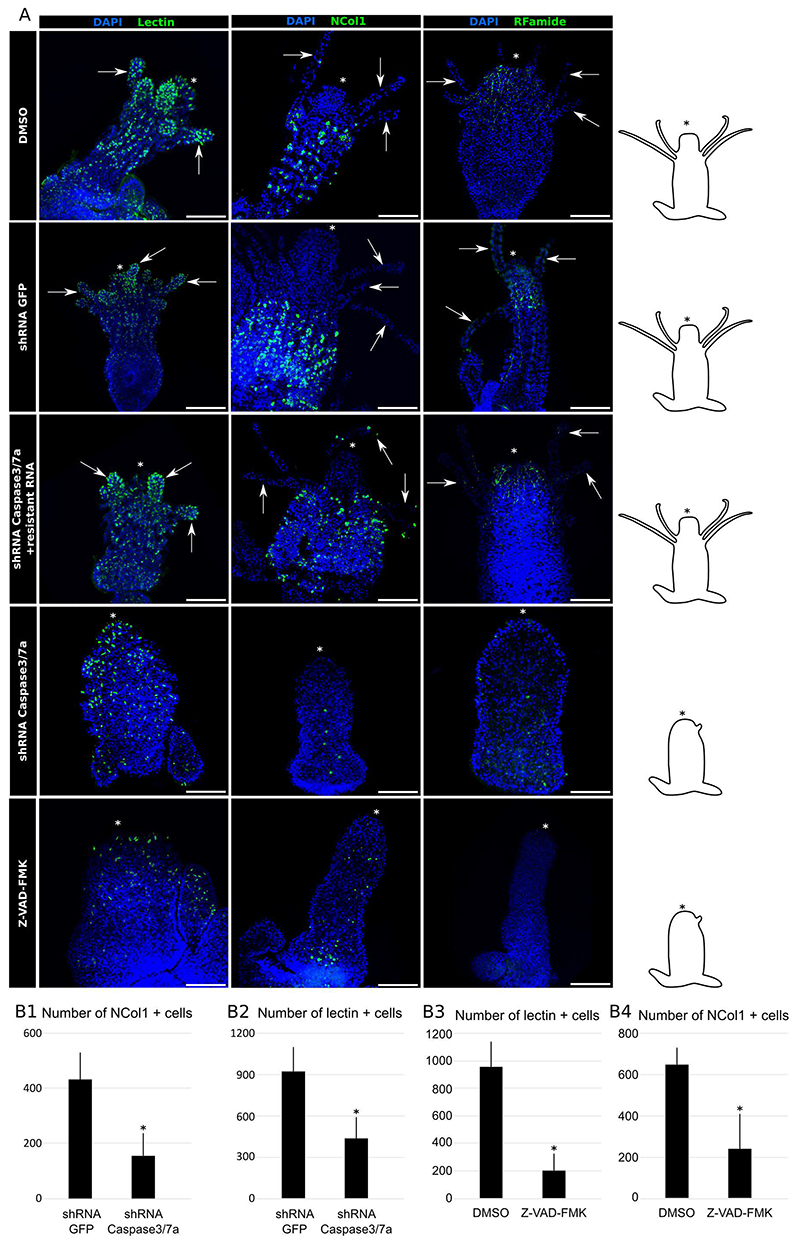
Head morphogenesis disruption leads to a primary polyp devoid of neurons. **A**, The numbers of both differentiating (NCol1) and fully differentiated nematocytes (lectin) were reduced in the Z-VAD-FMK treated and *Caspase 3/7a* disrupted larvae. Similarly, RFamide^+^ neurons were nearly absent when apoptosis was disrupted. Injection of the *Caspase 3/7a* resistant mRNA together with the shRNA rescued the phenotypes. **B**, Differences in NCol1^+^ (B1) and lectin^+^ (B2) cells numbers between shRNA GFP and shRNA *Caspase 3/7a* injected primary polyps are statistically significant. Similar effects are observed with Z-VAD-FMK treatment for both NCol1^+^ (B3) and lectin+ (B4) cells. Scale bar = 200µm. * in polyps images: oral pole. * in graph: p<0.05.

In apoptosis disrupted animals, we discovered a severe reduction in the numbers of Ncol1^+^ nematoblasts in the body column, and of lectin^+^ nematocytes in the body column and head of affected polyps post metamorphosis (Figure 3). Due to the abundance of lectin^+^ nematocytes in tentacles, we also compared their number in the body column only and found a significant decrease of their numbers (Figure S2). Similarly, the hypostome nerve net was underdeveloped and the number of neurons was close to zero in Z-VAD-FMK treated and *Caspase3/7a* shRNA-injected animals (Figure 3). Two scenarios could explain these outcomes: first, apoptosis signaling is essential for neurogenesis and tentacles development depends on neurons and/or nematocytes. Alternatively, apoptosis signaling could be essential for tentacle development and neurogenesis being compromised in their absence. A combination of the two, i.e., apoptosis being required for both head morphogenesis and neurogenesis, is also possible. Importantly, neurons seem to not be affected during larval development, as we detected a well-formed nerve net and numerous Ncol1^+^ cells in the gastrodermis of larvae injected with the *Caspase3/7a* shRNA before metamorphosis (Figure S3). This suggest that the effect of apoptosis inhibition on neurons is specific to metamorphosis.

Similar outcomes, with regards to nematogenesis, were shown previously. Inhibition of nematocyte differentiation by blocking Notch signaling, or by downregulating *Nanos2* (both *Notch and Nanos2* are expressed in nematoblasts), cause severe defects in tentacle morphogenesis (Gahan et al., 2017; Kanska and Frank, 2013; Quiroga-Artigas et al., 2020). Furthermore, the present study shows that inhibition of apoptosis causes severe reduction in the numbers of neurons and nematoblasts in the body column, which had normal morphology. Therefore, it is plausible that apoptosis signaling directly promotes neurogenesis/nematogenesis in *Hydractinia* metamorphosis. Importantly, co-injection of shRNA-resistant *Caspase3/7a* mRNA partially rescued the neural phenotype (Figure 3).

### TRAF genes are upregulated in the presence of apoptosis and are required for neurogenesis

**Figure 4:**
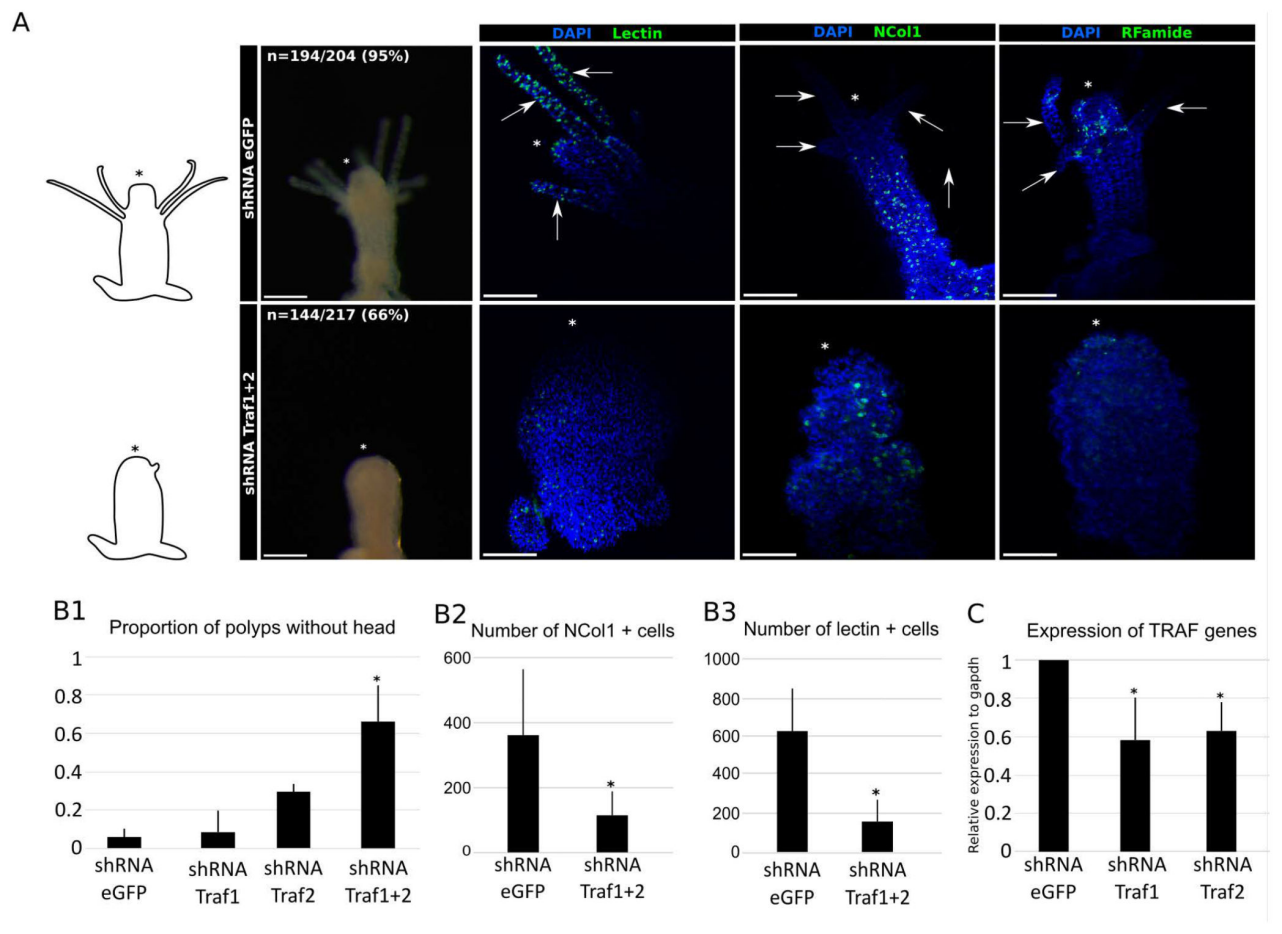
TRAF genes are involved in head morphogenesis during metamorphosis. **A**, Simultaneous *Traf1* and *Traf2* loss of function induced an underdeveloped head phenotype in primary polyps, and reduced number of nerve cells after metamorphosis. **B**, Effects of Traf genes loss of function on head development (B1), NCol1^+^ (B2) and lectin^+^ (B3) cells numbers are statistically significant. **C**, *Traf1* and *Traf2* shRNA efficiency was confirmed by real-time PCR in metamorphosing larvae. Scale bar = 200µm. * in polyps images: oral pole. * in graph: p<0.05.

To identify genes controlled by apoptosis during metamorphosis, we conducted a comparative transcriptomic analysis between control larvae, developed from embryos electroporated with shRNA against eGFP, and larvae developed from embryos electroporated with shRNA targeting *Caspase3/7a* to inhibit apoptosis at 5, 10, and 20 hpi. We identified a set of differentially expressed genes in apoptosis-inhibited animals (Figure S4, File S1). *Ncol1* expression at 20 hpi in apoptosis disrupted primary polyps was reduced, confirming our previous results (Figure S5). Next, we performed loss of functions by shRNA injection to target differentially expressed genes implicated in molecular pathways known to be involved in various morphogenetic processes in animals (Figure S6). In the cnidarian *Hydra*, Wnt signaling is emitted by apoptotic cells following bisection and is required for head regeneration (Chera et al., 2009). In tunicates, apoptosis-induced Wnt signaling is essential for siphon regeneration (Jeffery and Gorički, 2021). In our differential transcriptome, we detected only one Wnt (Wnt11b) that was downregulated in apoptosis-disrupted primary polyps (Figure S7). Thus, we choose to target Wnt11b (HyS0031.57), previously shown to be expressed in the polyp body column including the head (Hensel et al., 2014), and three Frizzled receptors that were also downregulated (HyS0011.93, HyS0030.175, HyS0072.42). Surprisingly, no visible phenotype was observed in any of these experiments (Figure S6). No other Wnt genes were downregulated when apoptosis was abolished. However, at 20 hpi, both Wnt1 and Wnt3 were upregulated (Figure S7). These genes are involved in the oral-aboral patterning of the body column during metamorphosis and regeneration. Their upregulation following apoptosis inhibition may represent a compensatory mechanism. Among our differential RNA sequencing we also found *NFκB* to be downregulated in apoptosis disrupted larvae. *NFκB* is known to be involved in neurogenesis and cell differentiation in mice (Denis-Donini et al., 2005; Zhang and Hu, 2012). However, we were unable to detect a function for *NFκB* (HyS0012.169) in *Hydractinia* metamorphosis (Figure S6). We identified two TRAF genes (hereafter named *Traf1* and *Traf2*) as the only ones downregulated at all three time points when apoptosis was inhibited. *Traf1* was reported to be expressed during *H. echinata* metamorphosis from settlement to primary polyp development (Mali and Frank, 2004), consistent with our transcriptomic analysis. We conducted loss of function experiments by shRNA injection to evaluate their function during metamorphosis. First, we confirmed *Traf1* and *Traf2* expression and the shRNA efficacy at 5 hpi by real-time PCR (Figure 4). Individual knockdown of each of the TRAF genes did not result in a strong effect; however, simultaneous loss of function by co-injection of the two shRNAs led to a phenotype similar to the one observed in apoptosis-inhibited larvae in terms of stunted tentacle development and lower numbers of nematoblasts, nematocytes, and neuron in the hypostome and body column (Figure 4). Therefore, *Traf1* and *Traf2* likely act redundantly to induce head morphogenesis and/or neurogenesis during metamorphosis. They could also act during larval development, but this has not been addressed here. TRAF genes are known activators of various pathways such as MAPK or NFκB (Shi and Sun, 2018). The MAPK pathway was reported to be required for neurogenesis in the cnidarian *Nematostella* (Layden et al., 2016) and regeneration of *Hydra* (Tursch et al., 2022).

### i-cell numbers are independent of apoptosis

**Figure 5:**
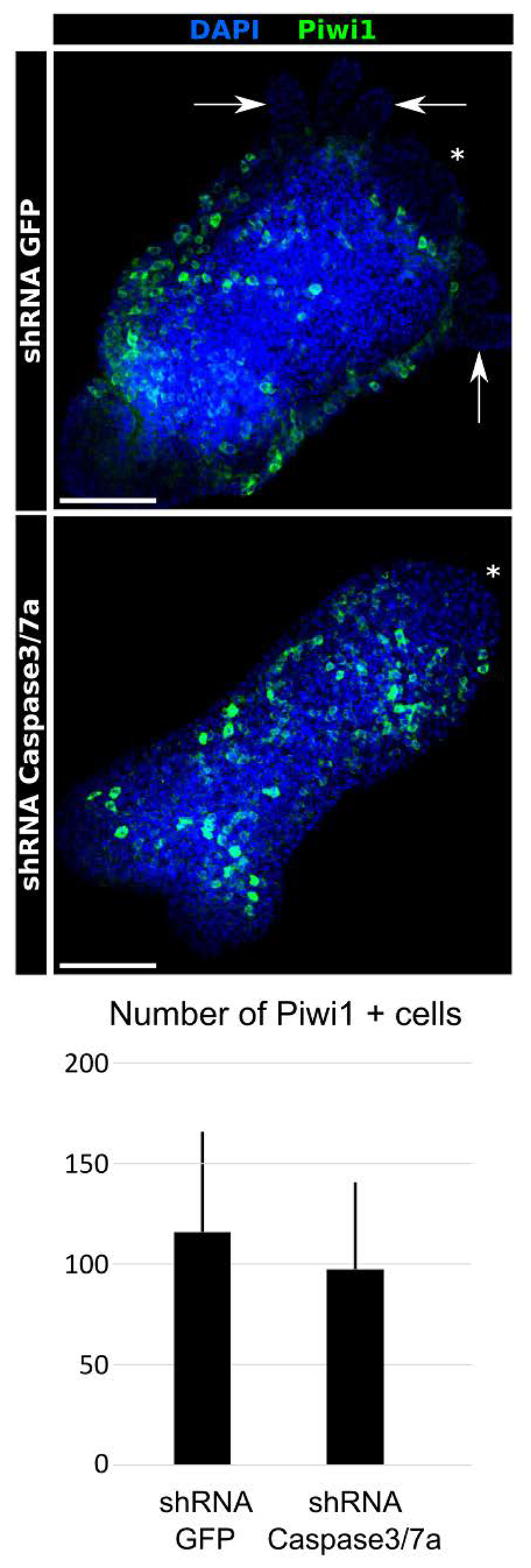
Apoptosis inhibition does not affect i-cell numbers. Piwi1 immunostaining of primary polyps showed a normal location and number of i-cells in the polyp epidermis in both control and apoptosis-inhibited animals. Difference in Piwi1 cell number is not statistically significant. *: oral pole. Scale bar = 200µm

In *Hydractinia*, all differentiated cells derive from a population of pluripotent stem cells called i-cells (Varley et al., 2023). These cells are mainly located in the epidermis of the polyp lower body column and in stolons, and marked by Piwi1 (Bradshaw et al., 2015; DuBuc et al., 2020). Defects in head morphogenesis could either be due to lack of i-cells or result from impaired differentiation into neurons and/or epithelial cells of the tentacles. To address this question, we conducted Piwi1 immunostaining to evaluate the numbers of i-cells in treated and control animals (Figure 5). We found that the i-cell population was unaffected in polyps that metamorphosed under apoptosis inhibition, suggesting that apoptosis is not required for i-cell self-renewal but may be implicated in their differentiation.

### Apoptosis is not required for *Hydractinia* head regeneration

**Figure 6:**
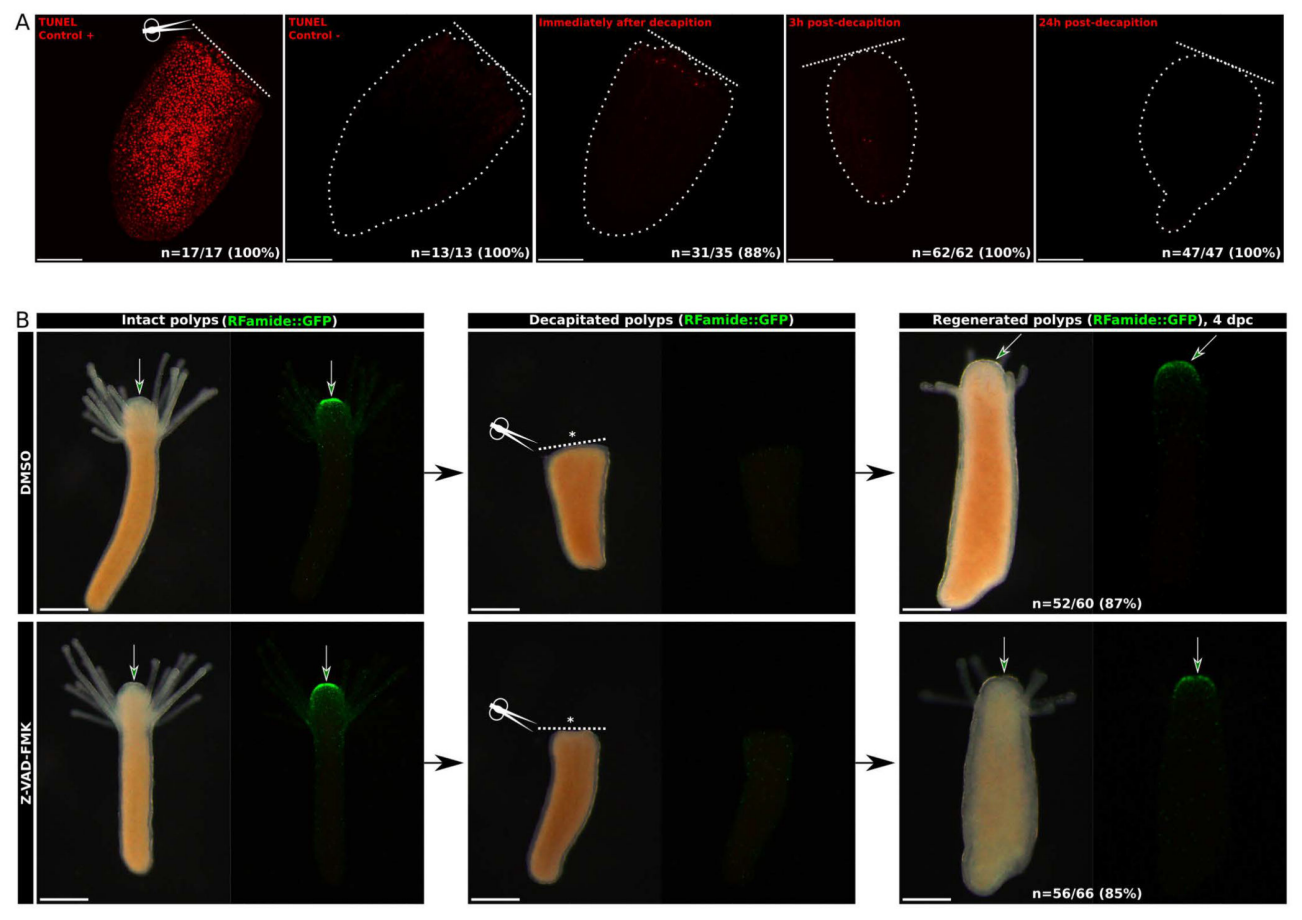
Apoptosis is not required for *Hydractinia* head regeneration. **A**, Only few TUNEL^+^nuclei were detected during the regeneration of the head at all time points considered. **B**, Follow up of decapitation and regeneration of feeding polyps from RFamide::eGFP transgenic line. Caspases are not required for head regeneration and nerve net formation of regenerating decapitated feeding polyps. The Z-VAD-FMK treatment did not affect the head regeneration as the tentacles and the mouth regenerate normally. Caspase inhibition did not prevent the nerve net establishment (arrows). Dpc, days post-decapitation. White dotted line: decapitation site. A, scale bar = 100µm. B, scale bar = 300µm. *: oral pole. White dotted line: decapitation plane.

In a related cnidarian, the freshwater polyp *Hydra*, apoptosis is required for i-cell proliferation during head regeneration (Chera et al., 2009). Given the importance of apoptosis for head development in metamorphosis, we aimed to investigate if a similar mechanism also acts during head regeneration in *Hydractinia*. Like *Hydra, Hydractinia* can regenerate a complete head within 3 days post amputation (Bradshaw et al., 2015). However, unlike in *Hydra*, we found that no significant apoptosis occurred during *Hydractinia* head regeneration. The number of TUNEL^+^ nuclei detected was insignificant (~10) at any stage of regeneration (Figure 6A). We exposed decapitated feeding polyps from the transgenic line *RFamide*::*GFP* that expresses GFP in neurons (Chrysostomou et al., 2022a) to Z-VAD-FMK and followed the regeneration process of the hypostome nerve net post decapitation. Consistent with the low number of apoptotic cells, Z-VAD-FMK-mediated inhibition of apoptosis did not affect the regeneration compared to control polyps. Treated and control regenerating polyps exhibited a normally appearing head, tentacles, and oral nervous system (Figure 6B). Therefore, we conclude that apoptosis and caspases are not involved in *Hydractinia* head morphogenesis during regeneration. This is surprising since involvement of apoptosis in regeneration was shown in various animals from distant phyla including ascidians, mice, and annelids (Fok et al., 2020; Jeffery and Gorički, 2021; Li et al., 2010). These data indicate that head morphogenesis in metamorphosis and regeneration involves distinct mechanisms.

In summary, a spatially and temporally regulated apoptotic wave plays a specific role in *Hydractinia* head development during metamorphosis but not during regeneration. It is executed by a single caspase (*Caspase3/7a*) and regulates neurogenesis and tentacle morphogenesis.

## Materials and methods

### *Hydractinia* husbandry

Adult *Hydractinia symbiolongicarpus* colonies were grown on glass slides in artificial seawater (ASW) at 18°C. Animals were fed four times per week with *Artemia franciscana* nauplii, and once a week with pureed oysters. To induce scheduled spawning, we kept the animals in a constant 14:10 light:dark cycle, where females and males spawn 1.5 hours after exposure to light. Metamorphosis was induced by cesium chloride incubation as previously described (Seipp et al., 2007).

### Gene identification and phylogenetic analysis

We performed Blastn and Blastx searches against the genome of *Hydractinia symbiolongicarpus* using the amino-acid sequences of human and *Hydra* caspases as queries followed by a reciprocal Blast search. We aligned amino-acid sequences using MAFFT 7 software and deleted the background with Gblocks 0.91b. Phylogenetic analysis was performed using Bayesian inference method with MrBayes 3.1.2 under mixed model. Analysis was run for 300,000 generations with 10 randomly started simultaneous Markov chains (first chain is a cold chain and the other ones are heated). One fourth of the topologies were discarded (burn-in values), and the remaining ones were used to calculate the posterior probability for nodes’ robustness.

### Pharmacological inhibition of apoptosis

Pan-caspase inhibitor Z-VAD-FMK (V116; Sigma-Aldrich) was resuspended in DMSO and used at a final concentration of 20 µM. Larvae were incubated in Z-VAD-FMK during the 3 hours before metamorphosis induction. Z-VAD-FMK was renewed at the metamorphosis induction time.

### Immunofluorescence and TUNEL staining

Larvae were fixed in 4% PFA in FSW overnight at 4°C and washed three times in PBS with 0.1% Tween (PBST). For long-term storage, samples were dehydrated by 10 minutes washes with gradual ethanol concentrations series in PBST (25%, 50%, 75% and 100% ethanol) and stored at -20°C. The dehydrated samples were then gradually rehydrated from ethanol to PBST by 10 minutes washes series. Samples were then washed in PBST and incubated for 3 hours in filtered 3% BSA in PBS 0.5%Triton. Primary antibody incubation was performed overnight at 4°C in 3% BSA in PBS 0.01%Triton while being rocked. Next, samples were washed three times for 30 minutes in PBS 0.01%Triton and blocked in 3% BSA/PBS 0.5%Triton/5% goat serum for 30 minutes at RT while being rocked. The secondary antibody was added in 3% BSA/0.5%Triton /5% goat serum for 90 minutes at RT. Samples were washed in PBST several times and their nuclei labeled using Hoechst 33258 (B2883 ; Sigma-Aldrich) for 45 minutes, and mounted in TDE.

TUNEL assays were performed using the TM red In Situ Cell Death Detection Kit (12156792910 ; Roche). Samples were fixed in 4% PFA in FSW for 90 minutes, washed three times with 1X PBS 0.01%Triton, permeabilized in 1X PBS 0.5%Triton for 90 minutes, and washed three times with 3% BSA in PBS. Samples were incubated at 37°C for 45 minutes in a 50 μL mix composed of 25 μL of reaction mix (Enzyme solution plus Label solution) and 25 μL of 3% BSA in PBS. Positive controls were first incubated for 25 minutes at 37°C in DNAse I solution (#EN0521 ; Thermo Fisher Scientific) and negative controls were incubated only in Label solution.

### Statistical analyses and imaging

Statistical significance was evaluated by Wilcoxon Mann-Whitney test using R 2.14.1. Effects were considered significant with a p-value <0.05 and denoted on graphs with an *. Images were taken with an Olympus Fluoview 1000 and 3000 confocal microscopes and analyzed using FIJI software.

### RNA synthesis, injection, and electroporation

Short-hairpin RNAs (shRNA) were designed as previously described (Chrysostomou et al., 2022a) and primers are provided in Table S2. Synthesis was done for three days using the HiScribe™ T7 High Yield RNA Synthesis Kit (E2040S; New England Biolabs). shRNAs were treated at RT for 1 hour with DNase I and finally purified using the Monarch® RNA Cleanup Kit (T2050L; New England Biolabs) according to manufacturer’s protocol. Embryos were injected with a mix composed of Dextran tracer with a final concentration of 2000 ng/µL of shRNA for caspases and each TRAF genes.

Electroporation mix was composed of 6 µL of 1.54M D-mannitol (240184; Merck) suspended in H_2_O, 9 µL of FSW containing eggs, and 9 µL of shRNA solution. Final concentration of shRNA was 1500 µg/µL. Electroporation was performed in Cuvettes Plus™ Electroporation Cuvettes (7321136 ; BTX) at 25V with a single 25 msec pulse using an in-house-made electroporator. Control larvae were injected/electroporated with a shRNA target eGFP m RNA.

Resistant *Caspase3/7a* sequence was ordered as gBlocks Gene Fragments from Integrated DNA Technologies. mRNA (Figure S1) was synthetized using the HiScribe™ T7 ARCA mRNA Kit (with tailing) (E2060S ; New England Biolabs) according to manufacturer’s instructions. Final concentration of resistant *Caspase3/7a* mRNA in injection mix was 500 ng/µL.

### RNA-seq libraries preparation, sequencing, and analysis

Control and treated metamorphosing larvae were sampled at 5, 10, and 20 hpi. Three biological replicates were done for each condition, making a total of 18 samples. Larvae were homogenized in TRIzol (15596026; Invitrogen) and stored at -80°C. RNA was extracted using homemade protocol (Chrysostomou et al., 2022a). The eluted RNA was quantified using NanoDrop and shipped to the Novogene Europe facility (Cambridge, UK) for further processing and sequencing. RNA integrity was assessed using the RNA Nano 6000 Assay Kit of the Bioanalyzer 2100 system (Agilent Technologies, CA, USA). Libraries were sequenced on an Illumina Novaseq platform and 150 bp paired-end reads were generated. Index of the reference genome (Kon-Nanjo et al., 2023) was built using Hisat2 v2.0.5 and paired-end clean reads were aligned to the reference genome using Hisat2 v2.0.5. The mapped reads of each sample were assembled by StringTie (v1.3.3b). Differential expression analysis of two conditions (three biological replicates per condition) was performed using the DESeq2 R package. The resulting P-values were adjusted using the Benjamini and Hochberg’s Approach, and genes with an adjusted P-value <=0.05 found by DESeq2 were designated as differentially expressed.

### Quantitative PCR

Larvae at 5 hpi (corresponding to our first differential RNA sequencing time point) were homogenized in TRIzol (15596026; Invitrogen) and RNA was extracted as previously described (Chrysostomou et al., 2022a). cDNAs were synthetized using the Omniscript RT Kit (205111 ; Qiagen) according to manufacturer’s protocol. Real-time PCR was performed with the TaqMan system (4444556 ; Applied Biosystems) on a StepOne Plus machine using the fast run under Quantification – Comparative Ct (ΔΔCt) experiment. Thermal cycler was running under the following profile: 95 °C for 15s for holding stage; 40 cycles of amplification with successive 95 °C for 1s and 64 °C for 30s with 10 µL of reaction mix. Real-time PCR experiments were done in triplicate using GAPDH as reference gene. Probes and primers are provided in Table S3.

## Supplementary Material

Fig S1

Fig S2

Fig S3

Fig S4

Fig S5

Fig S6

Fig S7

File S1

Supplementary Materials

## Data Availability

All data needed to evaluate the conclusions in this study are present in the paper and the supplementary materials. Sequences raw data are available under BioProject PRJNA961792. Any requests can be addressed to the corresponding authors.
