# Supplementary figures and images for: Apoptosis-dependent head development during metamorphosis of the cnidarian Hydractinia symbiolongicarpus
Source: Dev Biol. Author manuscript; Available in PMC 2025 Mar 15. (PMC7617490; doi:10.1016/j.ydbio.2024.08.010)

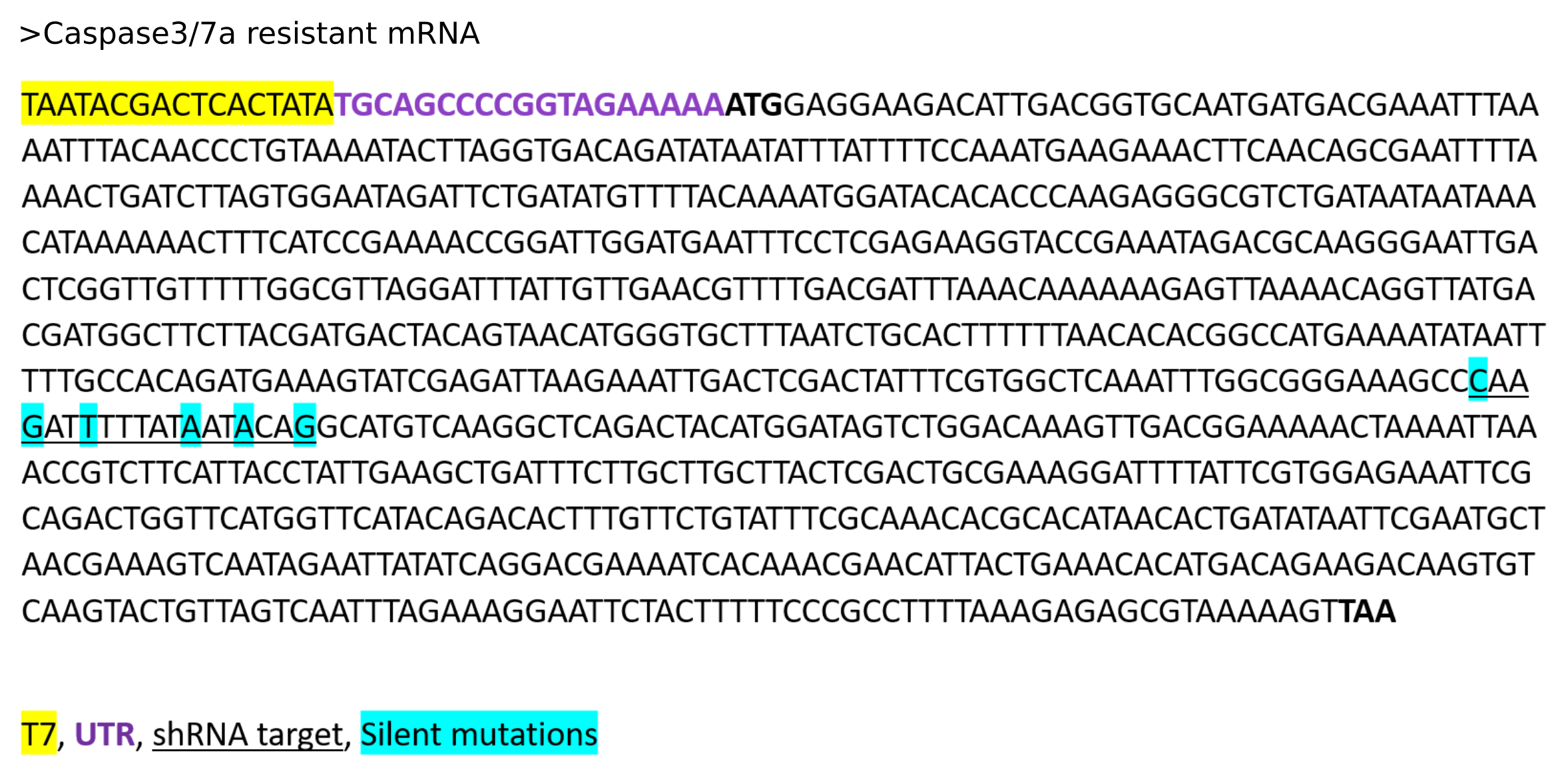

Supplement: Fig S1 [file EMS203329-supplement-Fig_S1.png]

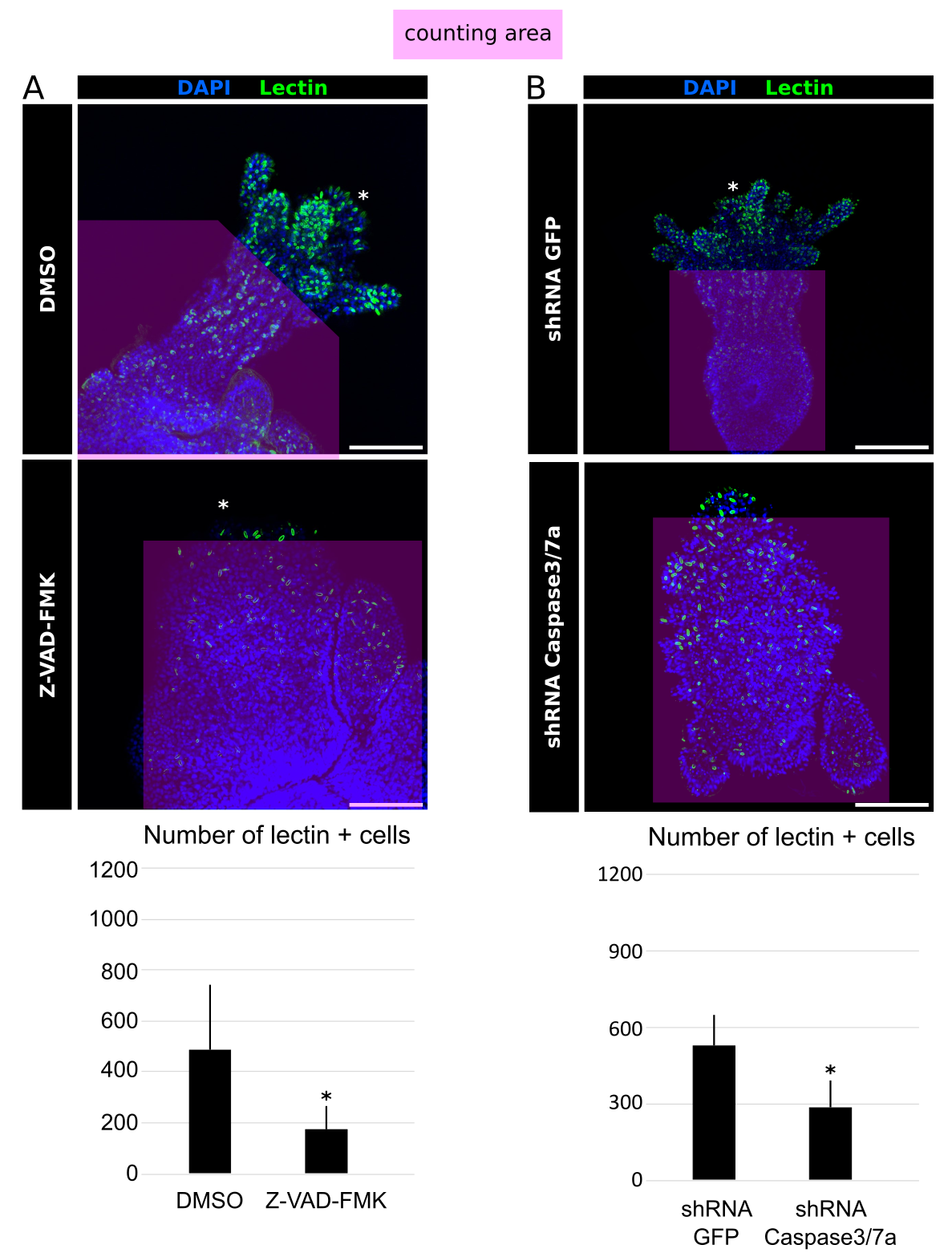

Supplement: Fig S2 [file EMS203329-supplement-Fig_S2.png]

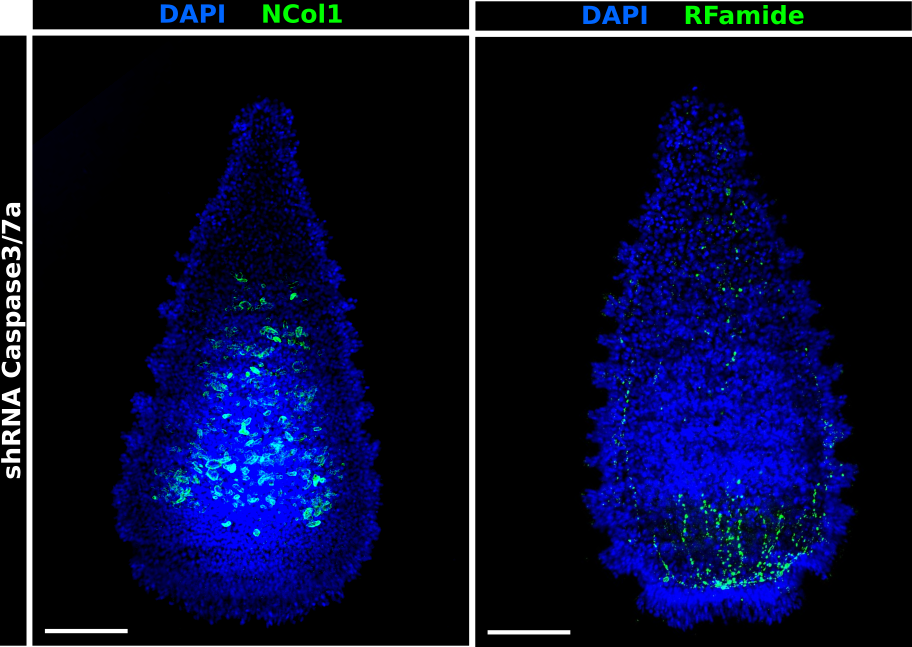

Supplement: Fig S3 [file EMS203329-supplement-Fig_S3.png]

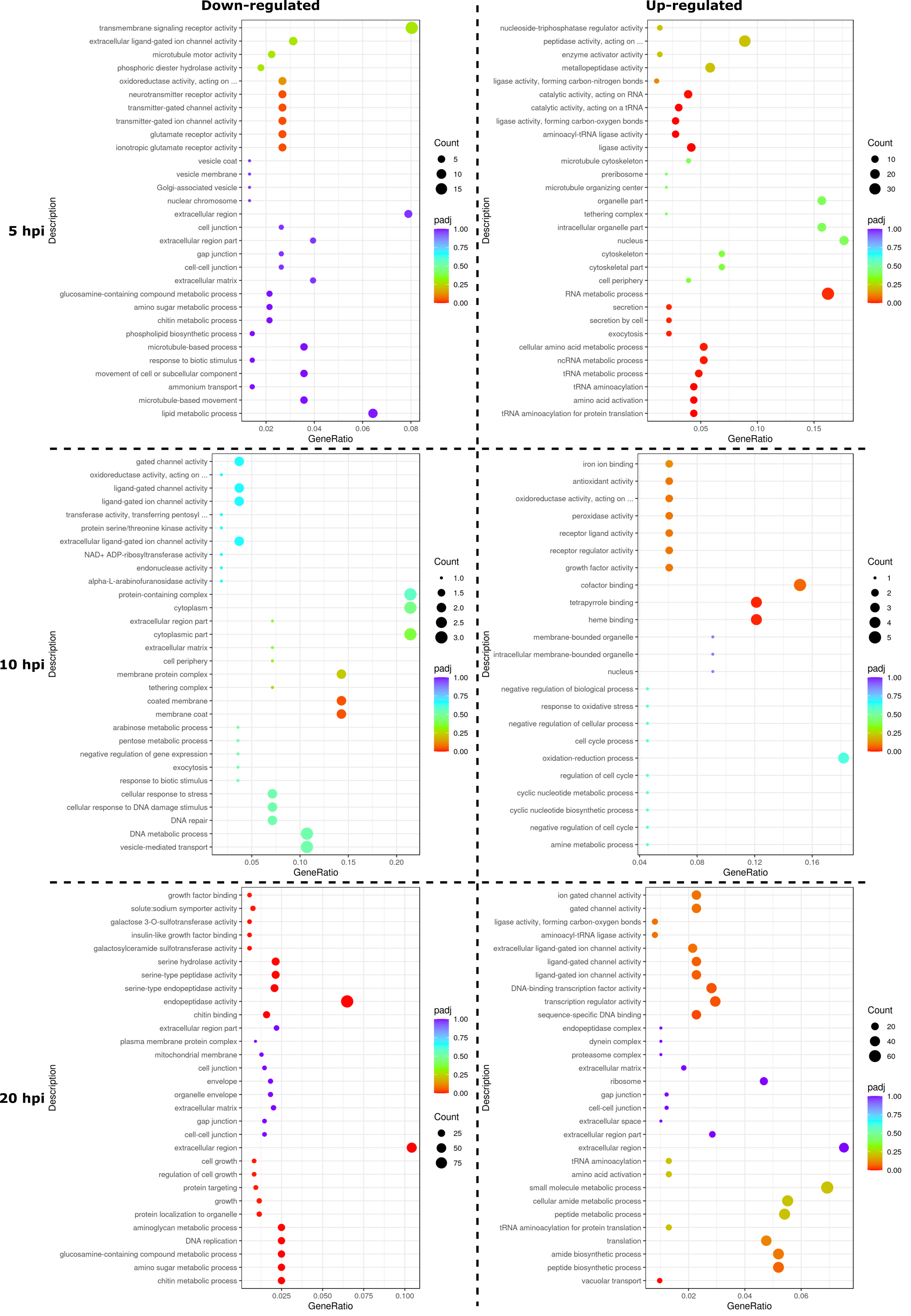

Supplement: Fig S4 [file EMS203329-supplement-Fig_S4.png]

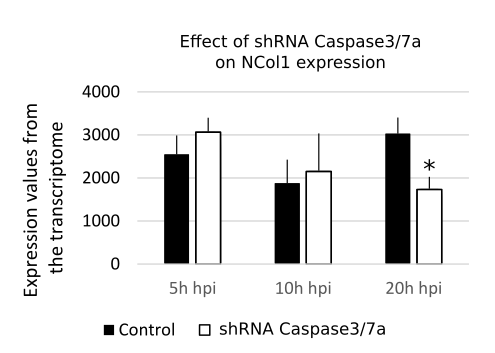

Supplement: Fig S5 [file EMS203329-supplement-Fig_S5.png]

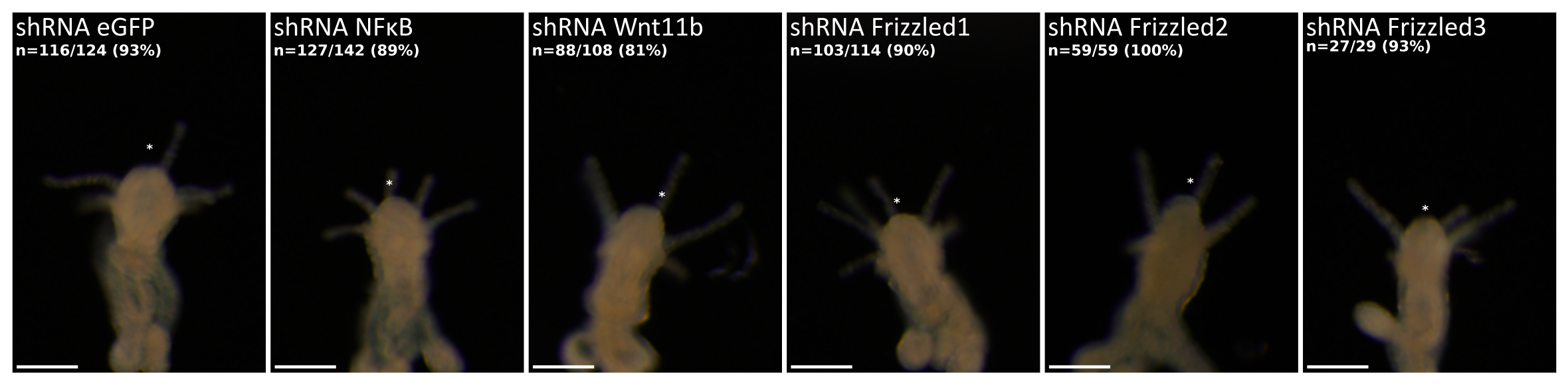

Supplement: Fig S6 [file EMS203329-supplement-Fig_S6.png]

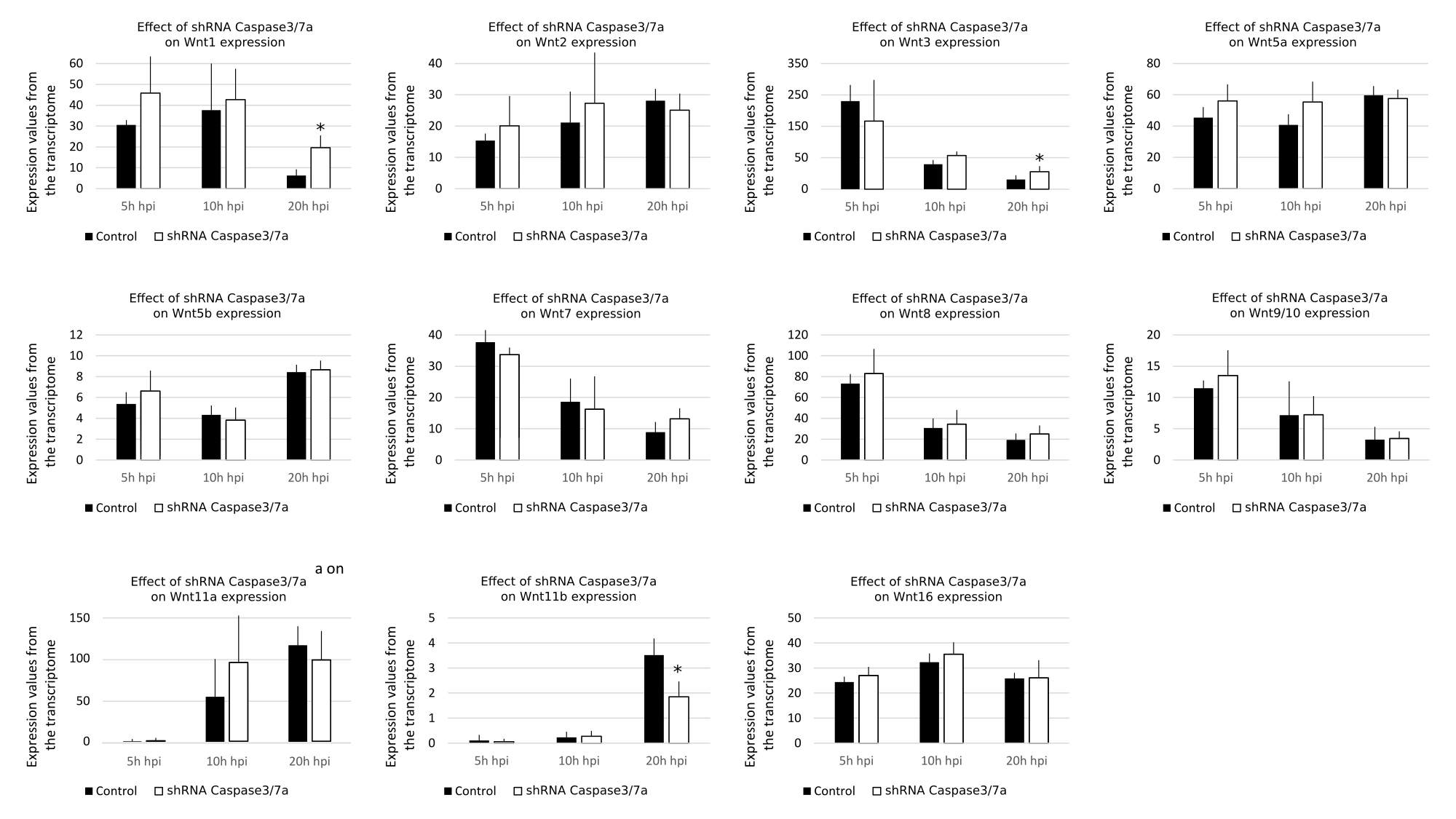

Supplement: Fig S7 [file EMS203329-supplement-Fig_S7.png]
