# Supplementary material for: Apoptosis-dependent head development during metamorphosis of the cnidarian Hydractinia symbiolongicarpus
Source: Dev Biol. Author manuscript; Available in PMC 2025 Mar 15. (PMC7617490; doi:10.1016/j.ydbio.2024.08.010)
Supplement: Supplementary Materials [file EMS203329-supplement-Supplementary_Materials.pdf]

Supplementary material legends

Figure S1. *Caspase3/7a mRNA* resistant to shRNA due to silent mutations on target site.

Figure S2. The inhibition of apoptosis, in addition to induce head morphogenesis disruption, resulted in a significant reduction of lectin$^+$ cells in the body column (magenta area). Scale bar = 200μm. * in polyps images: oral pole. * in graph: $p<0.05$.

Figure S3. 3 days old larvae injected with the *Caspase3/7a* shRNA. *Caspase3/7a* loss of function did not impair nerve net and neurons in larvae before metamorphosis. Scale bar = 100μm. * in polyps images: oral pole. * in graph: $p<0.05$.

Figure S4. Results of genes ontology enrichments from genes up- and down-regulated by apoptosis at 5, 10, and 20 hpi.

Figure S5. The expression of *Ncol1* is significantly downregulated at 20 hpi in larvae electroporated with the *Caspase3/7a* shRNA. Hpi: hours post-induction.

Figure S6: Loss of function of NFκB, Wnt11b, and Frizzled1 to 3 did not affect metamorphosis. *: oral pole. Scale bar = 200μm.

Figure S7. Expression of *Hydractinia Wnt* genes during metamorphosis of larvae electroporated with the *Caspase3/7a* shRNA. Among the expression profile, Wnt11b is downregulated at 20 hpi. Conversely, both Wnt1 and Wnt3 are up-regulated at 20 hpi. Hpi: hours post-induction.

Table S1: List of *Hydractinia symbiolongicarpus* Caspases with identification number from both available genome assemblies.

	https://research.nhgri.nih.gov/hydractinia/	Kon-Nanjo *et al*, 2023
Caspase3/7a	HyS0031.82 ; HyS0253.8	HSymV2.0_g03.04853_t1
Caspase3/7b	HyS0003.486	HSymV2.0_g09.17038_t1
Caspase3/7c	HyS0034.180	HSymV2.0_g14.24224 _t1
Caspase3/7d	HyS0074.45	HSymV2.0_g03.05227_t1
Caspase3/7e	HyS0012.28	HSymV2.0_g15.24941 _t1
Caspase1	HyS0118.8	HSymV2.0_g02.03211_t1
Caspase2	HyS0023.328	HSymV2.0_g13.23640_t1
Caspase8a	HyS0046.99	HSymV2.0_g10.17616_t1 ; HSymV2.0_g10.19163_t1
Caspase8b	HyS0024.235	HSymV2.0_g10.18231_t1

Table S2: list of shRNA.

Genes	shRNA
Caspase3/7a (HyS0253.8)	Fw: TAATACGACTCACTATAGAAAATAATTATCATTCAAATTTACTTTGAATGATAAATATTTTCTT<br>Rv: AAGAAAATATTTATCATTCAAAGTAAATTTGAATGATAATTATTTTCTATAGTGAGTCGTATTA
Caspase3/7b (HyS0003.486)	Fw: TAATACGACTCACTATAGGCAAAGCAATACGATTACCTTATTTACTAGGTAATCGTTTTGCTTTGCCTT<br>Rv: AAGGCAAAGCAAAACGATTACCTAGTAAATAAGGTAATCGTATTGCTTTGCCTATAGTGAGTCGTATTA
Caspase3/7c (HyS0034.180)	Fw: TAATACGACTCACTATAGCTTGTAGACGGAGAAAGTATATTTACTATACTTTCTCCCTCTACAAGCTT<br>Rv: AAGCTTGTAGAGGGAGAAAGTATAGTAAATATACTTTCTCCGTCTACAAGCTATAGTGAGTCGTATTA
Caspase3/7d (HyS0074.45)	Fw: TAATACGACTCACTATAGGTCCTGGTGGTCATCTCATTATTTACTAATGAGATGACGACCAGGACCTT<br>Rv: AAGGTCCTGGTCGTCATCTCATTAGTAAATAATGAGATGACCACCAGGACCTATAGTGAGTCGTATTA
Caspase1 (HyS0118.8)	Fw: TAATACGACTCACTATAGGTTCCCATCATGGCGTATTAATTTACTTAATACGCCATGTTGGGAACCTT<br>Rv: AAGGTTCCCAACATGGCGTATTAAGTAAATTAATACGCCATGATGGGAACCTATAGTGAGTCGTATTA
Traf1 (HyS0014.170)	Fw: TAATACGACTCACTATAGGAAGGAAGAATACCTTTATAATTTACTTATAAAGCTATTGTTCCTTCCTT<br>Rv: AAGGAAGGAACAATAGCTTTATAAGTAAATTATAAAGGTATTCTTCCTTCCTATAGTGAGTCGTATTA
Traf2 (HyS0003.508)	Fw: TAATACGACTCACTATAGGAGTGGCCTACATTGTGATTATTTACTAATCACAATGTACGCCACTCCTT<br>Rv: AAGGAGTGGCGTACATTGTGATTAGTAAATAATCACAATGTAGGCCACTCCTATAGTGAGTCGTATTA
eGFP	Fw: TAATACGACTCACTATAGTGAAGGTGATGCAACATATTCAAGAGATATGTTGCATCACCTTCACTT<br>Rv: AAGTGAAGGTGATGCAACATATCTCTTGAATATGTTGCATCACCTTCACTATAGTGAGTCGTATTA
Wnt11b (HyS0031.57)	Fw1: TAATACGACTCACTATAGGGAAGTTAAACGAGGTGAATATTTACTATTCACCTCGATTAACTTCCCTT<br>Rv1: AAGGGAAGTTAATCGAGGTGAATAGTAAATATTCACCTCGTTTAACTTCCCTATAGTGAGTCGTATTA<br>Fw2: TAATACGACTCACTATAGCGCTTGCTACTGGTGAAGAAATTTACTTTCTTCACCAGTACCAAGCGCTT<br>Rv2: AAGCGCTTGGTACTGGTGAAGAAAGTAAATTTCTTCACCAGTAGCAAGCGCTATAGTGAGTCGTATTA<br>Fw3: TAATACGACTCACTATAGGGCAAATTCGAGATTGTTCAATTTACTTGAACAATCTGGAATTTGCCCTT

	Rv3:<br>AAGGGCAAATTCCAGATTGTTCAAGTAAATTGAACAATCTCGAATTTGCCCTATAGTGAGTCGTATTA
Frizzled1 (HyS0011.93)	Fw:<br>TAATACGACTCACTATAGGAAGTTAACGCGAGGTCAAAATTTACTTTTGACCTGGCGTTTACTTCCTT<br>Rv:<br>AAGGAAGTAAACGCCAGGTCAAAAGTAAATTTTGACCTCGCGTTAACTTCCTATAGTGAGTCGTATTA
Frizzled2 (HyS0030.175)	Fw:<br>TAATACGACTCACTATAGGAGCAGGAAAAGAACTGAAAATTTACTTTTCAGTTCTATTCCTGCTCCTT<br>Rv:<br>AAGGAGCAGGAATAGAACTGAAAAGTAAATTTTCAGTTCTTTTCCTGCTCCTATAGTGAGTCGTATTA
Frizzled3 (HyS0072.42)	Fw:<br>TAATACGACTCACTATAGGCTTAATCCGACCGATCAAAATTTACTTTTGAACGGTCGGAATAAGCCTT<br>Rv:<br>AAGGCTTATTCCGACCGTTCAAAAGTAAATTTTGATCGGTCGGATTAAGCCTATAGTGAGTCGTATTA
NF-Kappa-B (HyS0012.169)	Fw:<br>TAATACGACTCACTATAGGCGAGTCCTCTTTAGAACTTATTTACTAAGTTCTAAAGACGACTCGCCTT<br>Rv:<br>AAGGCGAGTCGTCTTTAGAACTTAGTAAATAAGTTCTAAAGAGGACTCGCCTATAGTGAGTCGTATTA

Table S3: list of real-time PCR primers and probes.

Genes	Real-time PCR primers
Caspase3/7a<br>(HyS0253.8)	Fw: CCTCGAGAAGGTACCGAAATAG<br>Rv: TCGTAAGAAGCCATCGTCATAA<br>Probe: [6FAM] ACGCAAGGGAATTGACTCGGTTGT [TAMRA]
Traf1<br>(HyS0014.170)	Fw: GAGAAGTAACAGCGCTCCATAG<br>Rv: CCCATACCATCGCCGAATAG<br>Probe: [6FAM] TCGCTATGGTTACAAATTCTGCGCGA [TAMRA]
Traf2<br>(HyS0003.508)	Fw: CGGATGTTGATCGGAGATTGA<br>Rv: CTACCAGTGGTCGCATCTTT<br>Probe: [6FAM] TTAGAACGTGACGGTTTGCCGGAA [TAMRA]
GAPDH<br>(HyS0002.485)	Fw: CAAAGGCTGTTGGAAAGGTTATC<br>Rv: TCTTCAAACGCACTGTCAAATC<br>Probe: [6FAM] AGTGCCAGTTCCTGATGTTTCGGT [TAMRA]

Files S1. List of up and downregulated genes during metamorphosis between control larvae and apoptosis disrupted larvae at 5, 10, and 15 hours post-fertilization.
